# New Appliances and Things Medical

**Published:** 1897-12-04

**Authors:** 


### NEW APPLIANCES AND THINGS MEDICAL.

[We shall be glad to receive, at our Office, 28 & 29, Southampton Street, Strand, London, W.C., from the manufacturers, specimens of all new preparations and appliances which may be brought out from time to time.]

### MALT EXTRACT AND DIASTOL.

(Standard Malt Extract Company, London and Liverpool.)

We have on former occasions examined the preparations of this firm, and in June of 1895 we gave a careful analysis of the diastatic value. On further acquaintance with these preparations we feel all the more inclined to adhere to our original opinion that they are of first-rate quality. Practical experience has confirmed what the analysis of the laboratory demonstrated. Diastol, while possessing the digestive properties of the malt extract, must not be confounded with the latter product. The ferment has been extracted by a particular process, and exists in a highly concentrated and pure form.

### NEW PALATINOIDS.

(Oppenheimer, Son, and Co., Limited, London.)

Among the new palatinoids prepared by the above firm the following are some of the most useful. Ichthyol in three-grain doses. Owing to its nauseating taste and the large doses necessary, and the long continuance of the treatment which is frequently required, the possibility of prescribing the drug in the form of palatinoids will be much appreciated by physicians. Duodenin is another useful preparation now, thanks to Messrs. Oppenheimer, capable of administration in the form of palatinoids. Duodenin contains the ferments which are natural to the duodenum, namely, those of an amylolytic, proteolytic, and oleolytic action. In certain cases of deficient digestion duodenin may be found of signal advantage. Peptonate of iron with manganese sulphate, often found of advantage in the treatment of anæmia and kindred diseases, administered in the form of palatinoids, will be found far more agreeable than the highly unpleasant liquid form usually employed. Salol in the form of fine powder, and encapsuled with a casing of soluble glycerine jujube, is an excellent form for administration; this is undoubtedly a useful addition to the list of palatinoids. Palatinoids equivalent to half a drachm and Parrish's food in moderate doses will also be found useful.

### INVALID PORT AND WHISKEY.

(Washbourn Brothers, Bell Lane, Gloucester.)

The sample of port which we have received from the above firm appeals to us as a sound nutty wine, matured in the wood. Though sufficiently dry for invalid purposes, it is sweet enough to please the taste of those who are not used to the dry vintage wines of great age. For the price (38s. per dozen) it would be difficult to find a better all-round wine for invalids and convalescents. The whiskey, which is a Scotch blend, appears to be a spirit of good quality, well matured, mellow, and free from the so-called fusel oils. We recommend it to the notice of the rheumatic and gouty. The price is 48s. per dozen.

